# μ-Oxido-bis­{bis­[(penta­fluoro­phen­yl)methano­lato](η^5^-penta­methyl­cyclo­penta­dien­yl)titanium(IV)}

**DOI:** 10.1107/S1600536811027814

**Published:** 2011-07-16

**Authors:** Junseong Lee, Youngjo Kim

**Affiliations:** a Department of Chemistry, Chonnam National University, Gwangju 500-757, Republic of Korea; b Department of Chemistry, Chungbuk National University, Cheongju, Chungbuk 361-763, Republic of Korea

## Abstract

The dinuclear title complex, [Ti_2_(C_10_H_15_)_2_(C_7_H_2_F_5_O)_4_O], features two Ti^IV^ atoms bridged by an O atom. Each Ti atom is bonded to a η^5^-penta­methyl­cyclo­penta­dienyl ring, two (penta­fluoro­phen­yl)methano­late anions and to the bridging O atom. The environment around each Ti atom can be considered as a distorted tetra­hedron.

## Related literature

For related titanium complexes, Cp*Ti(OCH_2_C_6_F_5_)_3_ and Cp*Ti(OC_6_F_5_)_3_, see: Lee *et al.* (2007 ▶). For other related structures, see: Gowik *et al.* (1990 ▶); Thewalt & Schomburg (1977 ▶). For the use of dinuclear titanium complexes containing a cyclo­penta­dienyl ligand in organometallic catalysis, see: Noh *et al.* (2006 ▶); Wu *et al.* (2009 ▶); Yoon *et al.* (2011 ▶).
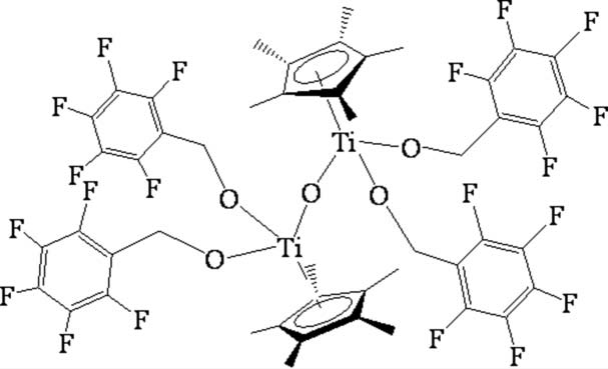


## Experimental

### 

#### Crystal data

[Ti_2_(C_10_H_15_)_2_(C_7_H_2_F_5_O)_4_O]
*M* *_r_* = 1170.58
Monoclinic, 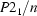

*a* = 11.371 (2) Å
*b* = 16.113 (3) Å
*c* = 27.340 (6) Å
β = 90.75 (3)°
*V* = 5008.8 (17) Å^3^
*Z* = 4
Mo *K*α radiation
μ = 0.44 mm^−1^
*T* = 293 K
0.15 × 0.12 × 0.10 mm

#### Data collection

Bruker SMART 1K CCD diffractometer
Absorption correction: multi-scan (*SADABS*; Bruker, 2004 ▶) *T*_min_ = 0.94, *T*_max_ = 0.96
28465 measured reflections
11233 independent reflections
5333 reflections with *I* > 2σ(*I*)
*R*_int_ = 0.042

#### Refinement

*R*[*F* ^2^ > 2σ(*F* ^2^)] = 0.075
*wR*(*F* ^2^) = 0.221
*S* = 1.03
11233 reflections
686 parameters
H-atom parameters constrained
Δρ_max_ = 0.33 e Å^−3^
Δρ_min_ = −0.37 e Å^−3^

### 

Data collection: *SMART* (Bruker, 2004 ▶); cell refinement: *SAINT* (Bruker, 2004 ▶); data reduction: *SAINT*; program(s) used to solve structure: *SHELXS97* (Sheldrick, 2008 ▶); program(s) used to refine structure: *SHELXL97* (Sheldrick, 2008 ▶); molecular graphics: *ORTEP-3* (Farrugia, 1997 ▶); software used to prepare material for publication: *SHELXTL* (Sheldrick, 2008 ▶).

## Supplementary Material

Crystal structure: contains datablock(s) I, global. DOI: 10.1107/S1600536811027814/lr2018sup1.cif

Structure factors: contains datablock(s) I. DOI: 10.1107/S1600536811027814/lr2018Isup2.hkl

Supplementary material file. DOI: 10.1107/S1600536811027814/lr2018Isup3.cdx

Additional supplementary materials:  crystallographic information; 3D view; checkCIF report

## Figures and Tables

**Fig. 1.**
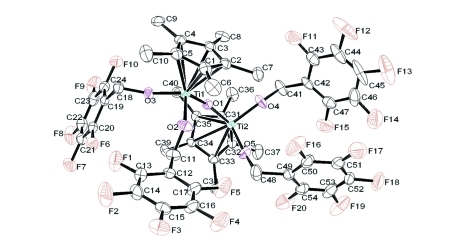
The molecular structure of the title compound (I). Displacement ellipsoids are drawn at the 15% probability level. H atoms are omitted for clarity.

**Fig. 2.**
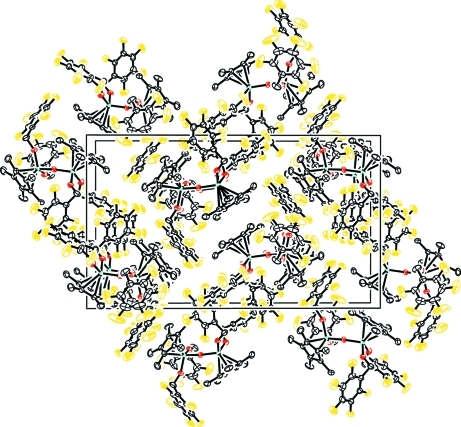
A packing diagram of compound (I). H atoms are omitted for clarity.

## supplementary crystallographic information

### Comment

Dinuclear titanium complexes containing a cyclopentadienyl ligand have attracted considerable attention in the fields of organometallic catalysis (Noh *et al.*, 2006; Wu *et al.*, 2009; Yoon *et al.*, 2011). Recently, we have reported the facile synthesis of Cp^*^Ti(OCH_2_C_6_F_5_)_3_ and Cp^*^Ti(OC_6_F_5_)_3_ (Cp^*^ = η^5^-pentamethylcyclopentadienyl). (Lee *et al.*, 2007) In continuation of our systematic studies on bimetallic pentamethylcyclopentadienyltitanium derivative using previously synthesized Cp^*^Ti(OCH_2_C_6_F_5_)_3_, the title complex (I) has been investigated.

The title compound (I) is the main product of the reaction of Cp^*^Ti(OCH_2_C_6_F_5_)_3_ with water in dichloromethane solution. In (I) (Fig. 1), the dinuclear structure shows two Ti atoms bridged by an oxygen atom, with approximately C_2_ symmetry. The Ti—C and Ti—O distances are in the range of 2.345 (5) - 2.400 (5) Å and 1.795 (3) - 1.819 (4) Å, respectively. The Ti—O—Ti angle of 163.1 (2) ° falls within the observed range (154 - 180°) for the previous reported compounds (Wu *et al.*, 2009; Thewalt & Schomburg, 1977; Gowik *et al.*, 1990). The dihedral angle between the pentamethylcyclopentadienyl rings is 38.3 (3) ° . In addition, three of the four dihedral angles between each Cp* ring and the benzene ring of the pentafluorobenzyloxy moieties attached to the same titanium atom are in the expected range, 72.4 (4)-78.0 (3)°.However, the remaining dihedral angle between Cp* ring (C1-C10) and benzene ring (C12-C17) is abnormally low, 5.6 (3)°.

### Experimental

Complex (I) was synthesized by hydrolysis of Cp^*^Ti(OCH_2_C_6_F_5_)_3_. Crystals were obtained by slow evaporation, in the refrigerator, using methylene chloride as solvent.

### Refinement

H atoms were positioned geometrically and refined using a riding model, with C—H = 0.93–0.97Å and with *U*_iso_(H) = 1.2 (1.5 for methyl groups) times *U*_eq_(C).

### Crystal data

**Table:**

[Ti_2_(C_10_H_15_)_2_(C_7_H_2_F_5_O)_4_O]	*F*(000) = 2360
*M**_r_* = 1170.58	*D*_x_ = 1.552 Mg m^−^^3^
Monoclinic, *P*2_1_/*n*	Mo *K*α radiation, λ = 0.71073 Å
*a* = 11.371 (2) Å	Cell parameters from 11233 reflections
*b* = 16.113 (3) Å	θ = 1.5–28.4°
*c* = 27.340 (6) Å	µ = 0.44 mm^−^^1^
β = 90.75 (3)°	*T* = 293 K
*V* = 5008.8 (17) Å^3^	Block, yellow
*Z* = 4	0.15 × 0.12 × 0.10 mm

### Data collection

**Table:**

Bruker SMART 1K CCD diffractometer	11233 independent reflections
Radiation source: fine-focus sealed tube	5333 reflections with *I* > 2σ(*I*)
graphite	*R*_int_ = 0.042
profile data from /ω scans	θ_max_ = 28.4°, θ_min_ = 1.5°
Absorption correction: multi-scan (*SADABS*; Bruker, 2004)	*h* = −14→14
*T*_min_ = 0.94, *T*_max_ = 0.96	*k* = −20→18
28465 measured reflections	*l* = −35→36

### Refinement

**Table:**

Refinement on *F*^2^	Primary atom site location: structure-invariant direct methods
Least-squares matrix: full	Secondary atom site location: difference Fourier map
*R*[*F*^2^ > 2σ(*F*^2^)] = 0.075	Hydrogen site location: inferred from neighbouring sites
*wR*(*F*^2^) = 0.221	H-atom parameters constrained
*S* = 1.03	*w* = 1/[σ^2^(*F*_o_^2^) + (0.0816*P*)^2^ + 3.8803*P*] where *P* = (*F*_o_^2^ + 2*F*_c_^2^)/3
11233 reflections	(Δ/σ)_max_ = 0.093
686 parameters	Δρ_max_ = 0.33 e Å^−^^3^
0 restraints	Δρ_min_ = −0.37 e Å^−^^3^

### Special details

**Table:**

Geometry. All e.s.d.'s (except the e.s.d. in the dihedral angle between two l.s. planes) are estimated using the full covariance matrix. The cell e.s.d.'s are taken into account individually in the estimation of e.s.d.'s in distances, angles and torsion angles; correlations between e.s.d.'s in cell parameters are only used when they are defined by crystal symmetry. An approximate (isotropic) treatment of cell e.s.d.'s is used for estimating e.s.d.'s involving l.s. planes.
Refinement. Refinement of *F*^2^ against ALL reflections. The weighted *R*-factor *wR* and goodness of fit *S* are based on *F*^2^, conventional *R*-factors *R* are based on *F*, with *F* set to zero for negative *F*^2^. The threshold expression of *F*^2^ > σ(*F*^2^) is used only for calculating *R*-factors(gt) *etc*. and is not relevant to the choice of reflections for refinement. *R*-factors based on *F*^2^ are statistically about twice as large as those based on *F*, and *R*- factors based on ALL data will be even larger.

### Fractional atomic coordinates and isotropic or equivalent isotropic displacement parameters (Å^2^)

**Table:**

	*x*	*y*	*z*	*U*_iso_*/*U*_eq_	
Ti1	0.69905 (7)	0.70860 (5)	0.55446 (3)	0.0610 (2)	
O2	0.8245 (4)	0.7653 (2)	0.52971 (14)	0.1012 (12)	
O3	0.5859 (3)	0.78829 (19)	0.55607 (12)	0.0817 (10)	
C1	0.7536 (6)	0.6170 (3)	0.48979 (19)	0.0849 (15)	
C2	0.7485 (5)	0.5714 (3)	0.53312 (18)	0.0754 (13)	
C3	0.6306 (5)	0.5716 (3)	0.54909 (18)	0.0737 (13)	
C4	0.5607 (5)	0.6174 (3)	0.5159 (2)	0.0789 (14)	
C5	0.6388 (6)	0.6466 (3)	0.47879 (19)	0.0864 (16)	
C6	0.8620 (7)	0.6320 (4)	0.4601 (2)	0.125 (2)	
H6A	0.8709	0.5880	0.4368	0.187*	
H6B	0.9297	0.6337	0.4814	0.187*	
H6C	0.8546	0.6840	0.4432	0.187*	
C7	0.8491 (6)	0.5271 (4)	0.5581 (2)	0.1062 (19)	
H7A	0.9218	0.5533	0.5496	0.159*	
H7B	0.8502	0.4701	0.5479	0.159*	
H7C	0.8393	0.5297	0.5929	0.159*	
C8	0.5845 (6)	0.5283 (3)	0.5939 (2)	0.108 (2)	
H8A	0.5724	0.4706	0.5868	0.162*	
H8B	0.5112	0.5531	0.6031	0.162*	
H8C	0.6405	0.5338	0.6203	0.162*	
C9	0.4290 (5)	0.6286 (4)	0.5177 (3)	0.116 (2)	
H9A	0.4085	0.6543	0.5481	0.175*	
H9B	0.3912	0.5755	0.5152	0.175*	
H9C	0.4036	0.6632	0.4910	0.175*	
C10	0.6041 (7)	0.6995 (4)	0.4354 (2)	0.128 (3)	
H10A	0.5255	0.7199	0.4396	0.192*	
H10B	0.6072	0.6667	0.4061	0.192*	
H10C	0.6573	0.7454	0.4329	0.192*	
C11	0.8684 (5)	0.8437 (4)	0.54140 (19)	0.0925 (17)	
H11A	0.9106	0.8415	0.5724	0.111*	
H11B	0.8039	0.8827	0.5445	0.111*	
C12	0.9504 (5)	0.8725 (3)	0.50152 (17)	0.0759 (14)	
C13	0.9147 (6)	0.9216 (4)	0.4638 (2)	0.0933 (16)	
C14	0.9891 (8)	0.9446 (4)	0.4263 (2)	0.106 (2)	
C15	1.1016 (7)	0.9178 (4)	0.4267 (2)	0.0913 (17)	
C16	1.1403 (6)	0.8705 (4)	0.4621 (2)	0.1034 (19)	
C17	1.0658 (6)	0.8480 (5)	0.4991 (2)	0.107 (2)	
C18	0.5031 (5)	0.8380 (3)	0.5324 (2)	0.0943 (18)	
H18A	0.4386	0.8040	0.5204	0.113*	
H18B	0.5387	0.8653	0.5047	0.113*	
C19	0.4570 (5)	0.9025 (3)	0.56781 (18)	0.0741 (13)	
C20	0.5065 (5)	0.9798 (3)	0.57017 (17)	0.0717 (13)	
C21	0.4703 (6)	1.0389 (3)	0.6014 (2)	0.0881 (16)	
C22	0.3829 (7)	1.0218 (5)	0.6323 (2)	0.108 (2)	
C23	0.3295 (6)	0.9449 (6)	0.6332 (3)	0.112 (2)	
C24	0.3685 (5)	0.8864 (4)	0.6001 (2)	0.0929 (17)	
F1	0.8044 (4)	0.9505 (3)	0.46415 (17)	0.1629 (18)	
F2	0.9501 (5)	0.9957 (4)	0.39154 (18)	0.204 (2)	
F3	1.1743 (4)	0.9407 (3)	0.39052 (12)	0.1376 (15)	
F4	1.2526 (4)	0.8409 (4)	0.46138 (18)	0.181 (2)	
F5	1.1099 (4)	0.7965 (4)	0.53375 (18)	0.198 (3)	
F6	0.5955 (3)	0.9989 (2)	0.54001 (12)	0.1019 (9)	
F7	0.5204 (4)	1.1147 (2)	0.60132 (13)	0.1307 (13)	
F8	0.3461 (5)	1.0802 (3)	0.66365 (16)	0.174 (2)	
F9	0.2437 (4)	0.9261 (4)	0.66401 (18)	0.179 (2)	
F10	0.3191 (3)	0.8103 (3)	0.60010 (17)	0.1395 (15)	
O1	0.7342 (3)	0.69325 (18)	0.61865 (9)	0.0667 (8)	
Ti2	0.81195 (7)	0.68839 (5)	0.67745 (3)	0.0597 (2)	
O4	0.8194 (4)	0.57809 (19)	0.69042 (12)	0.1022 (13)	
O5	0.9614 (3)	0.7123 (2)	0.66251 (14)	0.0936 (11)	
C31	0.6899 (5)	0.7044 (3)	0.74743 (17)	0.0774 (14)	
C32	0.8021 (5)	0.7342 (3)	0.76018 (17)	0.0824 (15)	
C33	0.8252 (5)	0.8052 (3)	0.7312 (2)	0.0831 (15)	
C34	0.7267 (4)	0.8178 (3)	0.69964 (16)	0.0660 (12)	
C35	0.6423 (4)	0.7559 (3)	0.71042 (16)	0.0694 (12)	
C36	0.6248 (6)	0.6332 (4)	0.7709 (2)	0.119 (2)	
H36A	0.5641	0.6547	0.7913	0.179*	
H36B	0.6788	0.6008	0.7903	0.179*	
H36C	0.5903	0.5988	0.7458	0.179*	
C37	0.8847 (7)	0.6981 (4)	0.7996 (2)	0.134 (3)	
H37A	0.9644	0.7011	0.7886	0.201*	
H37B	0.8642	0.6413	0.8056	0.201*	
H37C	0.8772	0.7295	0.8292	0.201*	
C38	0.9330 (5)	0.8588 (4)	0.7340 (3)	0.122 (2)	
H38A	0.9996	0.8257	0.7435	0.183*	
H38B	0.9215	0.9020	0.7577	0.183*	
H38C	0.9468	0.8832	0.7025	0.183*	
C39	0.7106 (5)	0.8856 (3)	0.66303 (19)	0.0887 (16)	
H39A	0.6527	0.9240	0.6745	0.133*	
H39B	0.6848	0.8623	0.6324	0.133*	
H39C	0.7839	0.9140	0.6587	0.133*	
C40	0.5229 (4)	0.7467 (4)	0.6860 (2)	0.0907 (16)	
H40A	0.4672	0.7818	0.7021	0.136*	
H40B	0.4976	0.6900	0.6882	0.136*	
H40C	0.5279	0.7624	0.6523	0.136*	
C41	0.8105 (7)	0.5068 (4)	0.7180 (2)	0.125 (3)	
H41A	0.7303	0.4863	0.7160	0.150*	
H41B	0.8283	0.5195	0.7519	0.150*	
C42	0.8924 (7)	0.4408 (3)	0.7007 (2)	0.0924 (18)	
C43	0.8583 (9)	0.3820 (5)	0.6683 (3)	0.122 (3)	
C44	0.9326 (15)	0.3222 (6)	0.6522 (4)	0.168 (6)	
C45	1.040 (2)	0.3138 (8)	0.6691 (6)	0.227 (12)	
C46	1.0770 (10)	0.3707 (8)	0.7006 (5)	0.167 (5)	
C47	1.0072 (9)	0.4328 (5)	0.7174 (3)	0.116 (2)	
C48	1.0701 (6)	0.7212 (4)	0.6445 (3)	0.131 (3)	
H48A	1.0995	0.7758	0.6535	0.157*	
H48B	1.0652	0.7190	0.6091	0.157*	
C49	1.1562 (5)	0.6578 (4)	0.6617 (2)	0.0864 (15)	
C50	1.1885 (6)	0.5919 (5)	0.6337 (3)	0.116 (2)	
C51	1.2669 (8)	0.5338 (6)	0.6502 (5)	0.147 (4)	
C52	1.3168 (7)	0.5416 (6)	0.6956 (6)	0.153 (5)	
C53	1.2846 (7)	0.6067 (9)	0.7226 (4)	0.147 (4)	
C54	1.2078 (6)	0.6612 (5)	0.7071 (3)	0.109 (2)	
F11	0.7497 (6)	0.3814 (3)	0.6495 (2)	0.188 (2)	
F12	0.9017 (10)	0.2631 (3)	0.61979 (19)	0.302 (6)	
F13	1.1190 (9)	0.2577 (4)	0.6560 (3)	0.299 (5)	
F14	1.1894 (7)	0.3709 (5)	0.7238 (3)	0.254 (4)	
F15	1.0435 (5)	0.4887 (3)	0.7512 (2)	0.183 (2)	
F16	1.1434 (4)	0.5844 (5)	0.5889 (2)	0.211 (3)	
F17	1.2997 (5)	0.4713 (4)	0.6225 (4)	0.292 (5)	
F18	1.3946 (4)	0.4883 (4)	0.7126 (4)	0.282 (5)	
F19	1.3326 (4)	0.6190 (5)	0.7685 (2)	0.236 (4)	
F20	1.1797 (4)	0.7275 (4)	0.7355 (2)	0.189 (2)	

### Atomic displacement parameters (Å^2^)

**Table:**

	*U*^11^	*U*^22^	*U*^33^	*U*^12^	*U*^13^	*U*^23^
Ti1	0.0713 (5)	0.0558 (5)	0.0559 (4)	−0.0022 (4)	0.0000 (4)	−0.0057 (3)
O2	0.117 (3)	0.088 (3)	0.100 (3)	−0.030 (2)	0.037 (2)	−0.002 (2)
O3	0.097 (2)	0.0652 (19)	0.083 (2)	0.0188 (18)	−0.0219 (18)	−0.0168 (16)
C1	0.107 (5)	0.081 (3)	0.067 (3)	0.011 (3)	0.006 (3)	−0.016 (3)
C2	0.092 (4)	0.058 (3)	0.076 (3)	0.006 (3)	−0.004 (3)	−0.015 (2)
C3	0.091 (4)	0.056 (3)	0.073 (3)	−0.007 (3)	−0.007 (3)	−0.011 (2)
C4	0.083 (4)	0.068 (3)	0.086 (4)	−0.002 (3)	−0.010 (3)	−0.029 (3)
C5	0.126 (5)	0.068 (3)	0.065 (3)	0.010 (3)	−0.015 (3)	−0.021 (2)
C6	0.148 (6)	0.123 (5)	0.104 (5)	0.011 (5)	0.052 (5)	−0.014 (4)
C7	0.120 (5)	0.086 (4)	0.111 (5)	0.029 (4)	−0.011 (4)	−0.004 (3)
C8	0.134 (5)	0.078 (4)	0.112 (5)	−0.036 (4)	0.013 (4)	−0.006 (3)
C9	0.094 (5)	0.093 (4)	0.162 (6)	−0.006 (4)	−0.030 (4)	−0.049 (4)
C10	0.206 (8)	0.106 (5)	0.072 (4)	0.038 (5)	−0.032 (4)	−0.015 (3)
C11	0.103 (4)	0.102 (4)	0.073 (3)	−0.027 (3)	0.015 (3)	−0.004 (3)
C12	0.091 (4)	0.072 (3)	0.065 (3)	−0.021 (3)	−0.001 (3)	0.002 (2)
C13	0.090 (4)	0.095 (4)	0.095 (4)	0.012 (3)	−0.003 (3)	0.005 (3)
C14	0.139 (6)	0.100 (4)	0.077 (4)	0.000 (4)	−0.013 (4)	0.032 (3)
C15	0.125 (5)	0.084 (4)	0.065 (3)	−0.032 (4)	0.008 (4)	0.003 (3)
C16	0.073 (4)	0.141 (6)	0.097 (4)	−0.020 (4)	−0.003 (3)	0.027 (4)
C17	0.085 (4)	0.149 (6)	0.086 (4)	−0.028 (4)	−0.015 (3)	0.045 (4)
C18	0.117 (5)	0.076 (3)	0.090 (4)	0.027 (3)	−0.035 (3)	−0.023 (3)
C19	0.082 (4)	0.063 (3)	0.076 (3)	0.012 (3)	−0.026 (3)	−0.002 (2)
C20	0.077 (3)	0.075 (3)	0.063 (3)	0.015 (3)	−0.009 (3)	−0.001 (2)
C21	0.117 (5)	0.071 (3)	0.077 (3)	0.015 (3)	−0.018 (3)	−0.011 (3)
C22	0.133 (6)	0.113 (5)	0.079 (4)	0.048 (5)	0.006 (4)	−0.010 (4)
C23	0.097 (5)	0.147 (7)	0.093 (5)	0.032 (5)	0.011 (4)	0.015 (5)
C24	0.075 (4)	0.092 (4)	0.112 (5)	−0.003 (3)	−0.013 (4)	0.019 (4)
F1	0.138 (4)	0.185 (4)	0.166 (4)	0.057 (3)	0.015 (3)	0.049 (3)
F2	0.241 (6)	0.220 (5)	0.152 (4)	0.044 (5)	0.006 (4)	0.114 (4)
F3	0.179 (4)	0.147 (3)	0.088 (2)	−0.053 (3)	0.040 (2)	0.011 (2)
F4	0.089 (3)	0.274 (6)	0.180 (4)	−0.008 (3)	0.018 (3)	0.079 (4)
F5	0.116 (3)	0.320 (7)	0.160 (4)	0.005 (4)	−0.004 (3)	0.145 (5)
F6	0.110 (2)	0.104 (2)	0.092 (2)	0.0002 (19)	0.0026 (19)	0.0058 (17)
F7	0.190 (4)	0.075 (2)	0.126 (3)	0.008 (2)	−0.026 (3)	−0.0211 (19)
F8	0.221 (5)	0.174 (4)	0.127 (3)	0.085 (4)	0.029 (3)	−0.041 (3)
F9	0.122 (3)	0.256 (6)	0.160 (4)	0.032 (4)	0.055 (3)	0.048 (4)
F10	0.110 (3)	0.122 (3)	0.185 (4)	−0.032 (2)	−0.029 (3)	0.031 (3)
O1	0.080 (2)	0.0655 (18)	0.0538 (16)	0.0010 (16)	−0.0096 (14)	−0.0077 (13)
Ti2	0.0718 (5)	0.0506 (4)	0.0566 (4)	0.0001 (4)	−0.0018 (4)	−0.0008 (3)
O4	0.176 (4)	0.0509 (19)	0.080 (2)	0.005 (2)	0.013 (2)	0.0099 (16)
O5	0.063 (2)	0.115 (3)	0.102 (3)	−0.003 (2)	−0.002 (2)	−0.004 (2)
C31	0.092 (4)	0.082 (3)	0.059 (3)	0.002 (3)	0.012 (3)	0.001 (2)
C32	0.106 (4)	0.082 (4)	0.059 (3)	0.020 (3)	−0.010 (3)	−0.016 (3)
C33	0.082 (4)	0.077 (3)	0.090 (4)	0.000 (3)	−0.005 (3)	−0.031 (3)
C34	0.074 (3)	0.057 (3)	0.067 (3)	0.005 (2)	0.001 (2)	−0.010 (2)
C35	0.067 (3)	0.080 (3)	0.061 (3)	0.009 (3)	0.005 (2)	−0.007 (2)
C36	0.138 (6)	0.120 (5)	0.100 (4)	0.015 (4)	0.046 (4)	0.030 (4)
C37	0.174 (7)	0.145 (6)	0.081 (4)	0.046 (5)	−0.052 (4)	−0.012 (4)
C38	0.096 (5)	0.093 (4)	0.177 (7)	−0.010 (4)	−0.026 (4)	−0.054 (4)
C39	0.119 (5)	0.057 (3)	0.090 (4)	0.014 (3)	0.003 (3)	−0.003 (2)
C40	0.071 (3)	0.102 (4)	0.099 (4)	0.003 (3)	0.003 (3)	−0.007 (3)
C41	0.194 (7)	0.071 (4)	0.112 (5)	0.021 (4)	0.045 (5)	0.031 (3)
C42	0.152 (6)	0.047 (3)	0.079 (4)	−0.004 (4)	0.021 (4)	0.018 (3)
C43	0.185 (9)	0.074 (5)	0.108 (5)	−0.005 (5)	0.003 (5)	0.032 (4)
C44	0.340 (19)	0.064 (5)	0.102 (6)	0.036 (9)	0.053 (9)	0.023 (4)
C45	0.42 (3)	0.079 (6)	0.181 (14)	0.107 (13)	0.170 (18)	0.049 (8)
C46	0.128 (8)	0.133 (9)	0.242 (13)	0.042 (7)	0.059 (8)	0.101 (9)
C47	0.155 (8)	0.081 (5)	0.113 (5)	−0.020 (5)	0.007 (5)	0.025 (4)
C48	0.106 (5)	0.121 (5)	0.167 (7)	0.028 (4)	0.044 (5)	0.057 (5)
C49	0.067 (3)	0.088 (4)	0.104 (4)	0.001 (3)	0.018 (3)	0.012 (3)
C50	0.070 (4)	0.134 (6)	0.144 (6)	0.011 (4)	−0.002 (4)	−0.025 (5)
C51	0.076 (5)	0.111 (6)	0.255 (12)	0.000 (5)	0.001 (6)	−0.041 (7)
C52	0.068 (5)	0.099 (6)	0.292 (15)	0.009 (5)	−0.003 (7)	0.060 (8)
C53	0.076 (5)	0.228 (12)	0.137 (7)	−0.024 (7)	−0.012 (5)	0.093 (8)
C54	0.076 (4)	0.127 (6)	0.123 (6)	−0.012 (4)	0.019 (4)	0.000 (5)
F11	0.253 (6)	0.120 (4)	0.188 (5)	−0.047 (4)	−0.073 (5)	0.051 (3)
F12	0.694 (18)	0.092 (3)	0.121 (4)	−0.030 (6)	0.017 (6)	−0.031 (3)
F13	0.437 (12)	0.187 (6)	0.277 (8)	0.160 (7)	0.208 (9)	0.088 (6)
F14	0.164 (6)	0.216 (7)	0.382 (11)	0.019 (5)	0.028 (6)	0.118 (7)
F15	0.235 (6)	0.135 (4)	0.178 (4)	−0.060 (4)	−0.047 (4)	0.035 (3)
F16	0.127 (4)	0.337 (8)	0.168 (4)	0.035 (4)	−0.018 (3)	−0.103 (5)
F17	0.138 (4)	0.182 (6)	0.557 (14)	0.023 (4)	0.039 (6)	−0.180 (7)
F18	0.104 (3)	0.214 (6)	0.527 (13)	0.029 (4)	−0.027 (5)	0.192 (8)
F19	0.110 (4)	0.446 (11)	0.151 (4)	−0.004 (5)	0.001 (3)	0.090 (6)
F20	0.130 (4)	0.260 (6)	0.177 (5)	−0.009 (4)	0.018 (3)	−0.088 (5)

### Geometric parameters (Å, °)

**Table:**

Ti1—O1	1.812 (3)	O1—Ti2	1.826 (3)
Ti1—O3	1.819 (3)	Ti2—O5	1.795 (4)
Ti1—O2	1.831 (4)	Ti2—O4	1.814 (3)
Ti1—C3	2.345 (5)	Ti2—C34	2.381 (4)
Ti1—C2	2.357 (5)	Ti2—C32	2.383 (5)
Ti1—C4	2.389 (5)	Ti2—C33	2.391 (5)
Ti1—C5	2.390 (5)	Ti2—C31	2.392 (5)
Ti1—C1	2.392 (5)	Ti2—C35	2.400 (5)
O2—C11	1.394 (6)	O4—C41	1.378 (6)
O3—C18	1.389 (6)	O5—C48	1.345 (7)
C1—C2	1.396 (7)	C31—C32	1.403 (7)
C1—C5	1.419 (8)	C31—C35	1.411 (6)
C1—C6	1.504 (8)	C31—C36	1.512 (7)
C2—C3	1.415 (7)	C32—C33	1.417 (7)
C2—C7	1.505 (7)	C32—C37	1.534 (7)
C3—C4	1.408 (7)	C33—C34	1.420 (7)
C3—C8	1.509 (7)	C33—C38	1.501 (8)
C4—C5	1.437 (8)	C34—C35	1.418 (6)
C4—C9	1.510 (8)	C34—C39	1.491 (6)
C5—C10	1.509 (7)	C35—C40	1.513 (7)
C6—H6A	0.9600	C36—H36A	0.9600
C6—H6B	0.9600	C36—H36B	0.9600
C6—H6C	0.9600	C36—H36C	0.9600
C7—H7A	0.9600	C37—H37A	0.9600
C7—H7B	0.9600	C37—H37B	0.9600
C7—H7C	0.9600	C37—H37C	0.9600
C8—H8A	0.9600	C38—H38A	0.9600
C8—H8B	0.9600	C38—H38B	0.9600
C8—H8C	0.9600	C38—H38C	0.9600
C9—H9A	0.9600	C39—H39A	0.9600
C9—H9B	0.9600	C39—H39B	0.9600
C9—H9C	0.9600	C39—H39C	0.9600
C10—H10A	0.9600	C40—H40A	0.9600
C10—H10B	0.9600	C40—H40B	0.9600
C10—H10C	0.9600	C40—H40C	0.9600
C11—C12	1.517 (7)	C41—H41A	0.9700
C11—H11A	0.9700	C41—H41B	0.9700
C11—H11B	0.9700	C41—C42	1.494 (8)
C11—C12	1.517 (7)	C42—C43	1.351 (9)
C12—C13	1.357 (7)	C42—C47	1.383 (10)
C12—C17	1.373 (8)	C43—F11	1.331 (9)
C13—F1	1.338 (7)	C43—C44	1.358 (14)
C13—C14	1.389 (8)	C44—C45	1.31 (2)
C14—F2	1.329 (7)	C44—F12	1.344 (13)
C14—C15	1.350 (9)	C45—C46	1.32 (2)
C15—C16	1.304 (8)	C45—F13	1.325 (13)
C15—F3	1.349 (6)	C46—C47	1.361 (13)
C16—F4	1.363 (7)	C46—F14	1.419 (14)
C16—C17	1.377 (8)	C47—F15	1.351 (8)
C17—F5	1.351 (7)	C48—C49	1.487 (8)
C18—C19	1.518 (7)	C48—H48A	0.9700
C18—H18A	0.9700	C48—H48B	0.9700
C18—H18B	0.9700	C49—C50	1.362 (9)
C19—C20	1.368 (7)	C49—C54	1.365 (9)
C19—C24	1.371 (8)	C50—F16	1.327 (8)
C20—C21	1.347 (7)	C50—C51	1.365 (12)
C20—F6	1.350 (6)	C51—F17	1.318 (9)
C21—C22	1.341 (9)	C51—C52	1.365 (14)
C21—F7	1.348 (6)	C52—F18	1.314 (9)
C22—F8	1.344 (7)	C52—C53	1.336 (14)
C22—C23	1.380 (10)	C53—C54	1.305 (11)
C23—F9	1.332 (8)	C53—F19	1.378 (10)
C23—C24	1.385 (9)	C54—F20	1.361 (8)
C24—F10	1.348 (7)		
			
O1—Ti1—O3	102.73 (14)	O5—Ti2—O4	102.31 (19)
O1—Ti1—O2	105.24 (17)	O5—Ti2—O1	103.83 (16)
O3—Ti1—O2	102.19 (18)	O4—Ti2—O1	103.58 (16)
O1—Ti1—C3	90.05 (15)	O5—Ti2—C34	105.03 (17)
O3—Ti1—C3	115.59 (18)	O4—Ti2—C34	145.72 (17)
O2—Ti1—C3	134.94 (18)	O1—Ti2—C34	89.59 (14)
O1—Ti1—C2	93.52 (16)	O5—Ti2—C32	101.9 (2)
O3—Ti1—C2	147.09 (18)	O4—Ti2—C32	96.93 (18)
O2—Ti1—C2	100.80 (19)	O1—Ti2—C32	142.57 (17)
C3—Ti1—C2	35.04 (17)	C34—Ti2—C32	57.50 (17)
O1—Ti1—C4	118.65 (18)	O5—Ti2—C33	85.32 (18)
O3—Ti1—C4	89.02 (17)	O4—Ti2—C33	130.43 (19)
O2—Ti1—C4	130.99 (19)	O1—Ti2—C33	122.23 (17)
C3—Ti1—C4	34.60 (17)	C34—Ti2—C33	34.61 (16)
C2—Ti1—C4	58.09 (18)	C32—Ti2—C33	34.54 (18)
O1—Ti1—C5	147.00 (17)	O5—Ti2—C31	136.03 (19)
O3—Ti1—C5	96.95 (18)	O4—Ti2—C31	88.59 (17)
O2—Ti1—C5	96.0 (2)	O1—Ti2—C31	114.87 (17)
C3—Ti1—C5	57.39 (18)	C34—Ti2—C31	57.29 (16)
C2—Ti1—C5	57.33 (18)	C32—Ti2—C31	34.17 (18)
C4—Ti1—C5	34.99 (18)	C33—Ti2—C31	57.06 (19)
O1—Ti1—C1	125.14 (17)	O5—Ti2—C35	139.11 (18)
O3—Ti1—C1	130.11 (18)	O4—Ti2—C35	113.96 (19)
O2—Ti1—C1	79.87 (19)	O1—Ti2—C35	85.94 (15)
C3—Ti1—C1	57.42 (19)	C34—Ti2—C35	34.51 (16)
C2—Ti1—C1	34.18 (17)	C32—Ti2—C35	57.00 (17)
C4—Ti1—C1	57.97 (19)	C33—Ti2—C35	57.13 (18)
C5—Ti1—C1	34.53 (18)	C31—Ti2—C35	34.24 (16)
C11—O2—Ti1	130.3 (3)	C41—O4—Ti2	156.9 (4)
C18—O3—Ti1	150.6 (3)	C48—O5—Ti2	169.8 (5)
C2—C1—C5	108.0 (5)	C32—C31—C35	108.4 (5)
C2—C1—C6	125.9 (6)	C32—C31—C36	127.1 (5)
C5—C1—C6	126.1 (6)	C35—C31—C36	124.4 (5)
C2—C1—Ti1	71.5 (3)	C32—C31—Ti2	72.6 (3)
C5—C1—Ti1	72.7 (3)	C35—C31—Ti2	73.2 (3)
C6—C1—Ti1	121.4 (4)	C36—C31—Ti2	123.3 (4)
C1—C2—C3	108.1 (5)	C31—C32—C33	108.2 (5)
C1—C2—C7	126.6 (5)	C31—C32—C37	126.2 (6)
C3—C2—C7	125.3 (5)	C33—C32—C37	125.6 (6)
C1—C2—Ti1	74.3 (3)	C31—C32—Ti2	73.3 (3)
C3—C2—Ti1	72.0 (3)	C33—C32—Ti2	73.0 (3)
C7—C2—Ti1	121.0 (3)	C37—C32—Ti2	120.9 (4)
C4—C3—C2	109.4 (5)	C32—C33—C34	107.8 (5)
C4—C3—C8	124.5 (5)	C32—C33—C38	126.4 (6)
C2—C3—C8	126.2 (5)	C34—C33—C38	125.8 (6)
C4—C3—Ti1	74.4 (3)	C32—C33—Ti2	72.4 (3)
C2—C3—Ti1	72.9 (3)	C34—C33—Ti2	72.3 (3)
C8—C3—Ti1	120.1 (3)	C38—C33—Ti2	122.0 (4)
C3—C4—C5	106.1 (5)	C35—C34—C33	107.7 (4)
C3—C4—C9	126.4 (6)	C35—C34—C39	125.3 (5)
C5—C4—C9	127.4 (6)	C33—C34—C39	127.0 (5)
C3—C4—Ti1	71.0 (3)	C35—C34—Ti2	73.5 (3)
C5—C4—Ti1	72.5 (3)	C33—C34—Ti2	73.1 (3)
C9—C4—Ti1	124.0 (3)	C39—C34—Ti2	121.1 (3)
C1—C5—C4	108.4 (5)	C31—C35—C34	108.0 (4)
C1—C5—C10	126.0 (6)	C31—C35—C40	126.3 (5)
C4—C5—C10	125.6 (6)	C34—C35—C40	125.7 (5)
C1—C5—Ti1	72.8 (3)	C31—C35—Ti2	72.6 (3)
C4—C5—Ti1	72.5 (3)	C34—C35—Ti2	72.0 (3)
C10—C5—Ti1	120.9 (4)	C40—C35—Ti2	120.8 (3)
C1—C6—H6A	109.5	C31—C36—H36A	109.5
C1—C6—H6B	109.5	C31—C36—H36B	109.5
H6A—C6—H6B	109.5	H36A—C36—H36B	109.5
C1—C6—H6C	109.5	C31—C36—H36C	109.5
H6A—C6—H6C	109.5	H36A—C36—H36C	109.5
H6B—C6—H6C	109.5	H36B—C36—H36C	109.5
C2—C7—H7A	109.5	C32—C37—H37A	109.5
C2—C7—H7B	109.5	C32—C37—H37B	109.5
H7A—C7—H7B	109.5	H37A—C37—H37B	109.5
C2—C7—H7C	109.5	C32—C37—H37C	109.5
H7A—C7—H7C	109.5	H37A—C37—H37C	109.5
H7B—C7—H7C	109.5	H37B—C37—H37C	109.5
C3—C8—H8A	109.5	C33—C38—H38A	109.5
C3—C8—H8B	109.5	C33—C38—H38B	109.5
H8A—C8—H8B	109.5	H38A—C38—H38B	109.5
C3—C8—H8C	109.5	C33—C38—H38C	109.5
H8A—C8—H8C	109.5	H38A—C38—H38C	109.5
H8B—C8—H8C	109.5	H38B—C38—H38C	109.5
C4—C9—H9A	109.5	C34—C39—H39A	109.5
C4—C9—H9B	109.5	C34—C39—H39B	109.5
H9A—C9—H9B	109.5	H39A—C39—H39B	109.5
C4—C9—H9C	109.5	C34—C39—H39C	109.5
H9A—C9—H9C	109.5	H39A—C39—H39C	109.5
H9B—C9—H9C	109.5	H39B—C39—H39C	109.5
C5—C10—H10A	109.5	C35—C40—H40A	109.5
C5—C10—H10B	109.5	C35—C40—H40B	109.5
H10A—C10—H10B	109.5	H40A—C40—H40B	109.5
C5—C10—H10C	109.5	C35—C40—H40C	109.5
H10A—C10—H10C	109.5	H40A—C40—H40C	109.5
H10B—C10—H10C	109.5	H40B—C40—H40C	109.5
O2—C11—C12	109.5 (4)	O4—C41—C42	111.7 (5)
O2—C11—H11A	109.8	O4—C41—H41A	109.3
C12—C11—H11A	109.8	C42—C41—H41A	109.3
O2—C11—H11B	109.8	O4—C41—H41B	109.3
C12—C11—H11B	109.8	C42—C41—H41B	109.3
H11A—C11—H11B	108.2	H41A—C41—H41B	107.9
C13—C12—C17	114.1 (5)	C43—C42—C47	114.4 (7)
C13—C12—C11	122.9 (6)	C43—C42—C41	122.1 (8)
C17—C12—C11	122.9 (5)	C47—C42—C41	123.5 (7)
F1—C13—C12	118.0 (6)	F11—C43—C42	121.0 (9)
F1—C13—C14	119.5 (6)	F11—C43—C44	116.6 (10)
C12—C13—C14	122.5 (6)	C42—C43—C44	122.4 (10)
F2—C14—C15	120.8 (7)	C45—C44—F12	113.3 (15)
F2—C14—C13	119.5 (7)	C45—C44—C43	122.8 (14)
C15—C14—C13	119.6 (5)	F12—C44—C43	123.8 (15)
C16—C15—F3	120.0 (7)	C44—C45—C46	116.3 (12)
C16—C15—C14	120.3 (6)	C44—C45—F13	127 (2)
F3—C15—C14	119.8 (6)	C46—C45—F13	116 (2)
C15—C16—F4	120.1 (6)	C45—C46—C47	123.4 (13)
C15—C16—C17	119.6 (6)	C45—C46—F14	124.5 (15)
F4—C16—C17	120.2 (6)	C47—C46—F14	112.0 (15)
F5—C17—C12	119.3 (5)	F15—C47—C46	123.1 (11)
F5—C17—C16	116.8 (6)	F15—C47—C42	116.2 (8)
C12—C17—C16	123.9 (5)	C46—C47—C42	120.6 (10)
O3—C18—C19	109.6 (4)	O5—C48—C49	114.5 (5)
O3—C18—H18A	109.7	O5—C48—H48A	108.6
C19—C18—H18A	109.7	C49—C48—H48A	108.6
O3—C18—H18B	109.7	O5—C48—H48B	108.6
C19—C18—H18B	109.7	C49—C48—H48B	108.6
H18A—C18—H18B	108.2	H48A—C48—H48B	107.6
C20—C19—C24	116.6 (5)	C50—C49—C54	115.2 (7)
C20—C19—C18	120.5 (5)	C50—C49—C48	122.6 (7)
C24—C19—C18	122.9 (5)	C54—C49—C48	122.2 (7)
C21—C20—F6	117.6 (5)	F16—C50—C49	119.1 (7)
C21—C20—C19	123.0 (6)	F16—C50—C51	119.0 (8)
F6—C20—C19	119.4 (5)	C49—C50—C51	121.9 (8)
C22—C21—C20	119.3 (6)	F17—C51—C50	121.6 (11)
C22—C21—F7	120.3 (6)	F17—C51—C52	118.4 (11)
C20—C21—F7	120.5 (6)	C50—C51—C52	119.9 (9)
C21—C22—F8	119.8 (8)	F18—C52—C53	120.5 (14)
C21—C22—C23	121.7 (6)	F18—C52—C51	122.1 (14)
F8—C22—C23	118.5 (8)	C53—C52—C51	117.4 (8)
F9—C23—C22	122.8 (8)	C54—C53—C52	122.4 (10)
F9—C23—C24	120.1 (8)	C54—C53—F19	117.1 (13)
C22—C23—C24	117.1 (6)	C52—C53—F19	120.5 (12)
F10—C24—C19	118.8 (6)	C53—C54—F20	120.2 (10)
F10—C24—C23	118.8 (7)	C53—C54—C49	123.1 (9)
C19—C24—C23	122.4 (6)	F20—C54—C49	116.7 (7)
Ti1—O1—Ti2	163.1 (2)		
